# A Co-driven Functional Electrical Stimulation Control Strategy by Dynamic Surface Electromyography and Joint Angle

**DOI:** 10.3389/fnins.2022.909602

**Published:** 2022-07-08

**Authors:** Rui Xu, Xinyu Zhao, Ziyao Wang, Hengyu Zhang, Lin Meng, Dong Ming

**Affiliations:** ^1^Laboratory of Motor Rehabilitation, Academy of Medical Engineering and Translational Medicine, Tianjin University, Tianjin, China; ^2^College of Precision Instruments and Optoelectronics Engineering, Tianjin University, Tianjin, China

**Keywords:** surface electromyography, functional electrical stimulation, joint torque control, polynomial fitting, kinematics

## Abstract

Functional electrical stimulation (FES) is widely used in neurorehabilitation to improve patients’ motion ability. It has been verified to promote neural remodeling and relearning, during which FES has to produce an accurate movement to obtain a good efficacy. Therefore, many studies have focused on the relationship between FES parameters and the generated movements. However, most of the relationships have been established in static contractions, which leads to an unsatisfactory result when applied to dynamic conditions. Therefore, this study proposed a FES control strategy based on the surface electromyography (sEMG) and kinematic information during dynamic contractions. The pulse width (PW) of FES was determined by a direct transfer function (DTF) with sEMG features and joint angles as the input. The DTF was established by combing the polynomial transfer functions of sEMG and joint torque and the polynomial transfer functions of joint torque and FES. Moreover, the PW of two FES channels was set based on the muscle synergy ratio obtained through sEMG. A total of six healthy right-handed subjects were recruited in this experiment to verify the validity of the strategy. The PW of FES applied to the left arm was evaluated based on the sEMG of the right extensor carpi radialis (ECR) and the right wrist angle. The coefficient of determination (*R*^2^) and the normalized root mean square error (NRMSE) of FES-included and voluntary wrist angles and torques were used to verify the performance of the strategy. The result showed that this study achieved a high accuracy (*R*^2^ = 0.965 and NRMSE = 0.047) of joint angle and a good accuracy (*R*^2^ = 0.701 and NRMSE = 0.241) of joint torque reproduction during dynamic movements. Moreover, the DTF in real-time FES system also had a nice performance of joint angle fitting (*R*^2^ = 0.940 and NRMSE = 0.071) and joint torque fitting (*R*^2^ = 0.607 and NRMSE = 0.303). It is concluded that the proposed strategy is able to generate proper FES parameters based on sEMG and kinematic information for dynamic movement reproduction and can be used in a real-time FES system combined with bilateral movements for better rehabilitation.

## Introduction

Functional electrical stimulation (FES) is widely used in neurorehabilitation such as the rehabilitation of spinal cord injury and stroke (Glanz et al., 1996). It applies an electric current to one or more muscles to stimulate its peripheral motor nerves, producing muscle contractions and prompting the limbs to complete corresponding functional motions (Lynch and Popovic, 2008). FES has been proven to be effective in improving upper extremity motor abilities in patients with stroke (Glanz et al., 1996; Chan et al., 2009; Knutson et al., 2016) and has been combined with rehabilitation robots and rehabilitation training games (Hodkin et al., 2018; Fu et al., 2020), as it promotes neural remodeling and relearning in the patients with nerve damage (Quandt and Hummel, 2014). At present, most practical applications of FES use preset parameters to stimulate muscles, i.e., a specific waveform, intensity, and frequency according to a predetermined procedure (Lynch and Popovic, 2008). Although the triggered FES can better promote nerve remodeling and improve the treatment effect due to the combination of the user’s active intention (Kimberley et al., 2004; Barsi et al., 2008), more biomimetic FES strategies may better facilitate the relearning process.

Functional electrical stimulation combined with bilateral movement (FES-BM) is one of the FES training methods that allows patients to actively participate. It allows the patients with hemiplegia to use the healthy side to control FES of the paralyzed side, and it can control not only the triggering but also the intensity of FES (Knutson et al., 2007). It was reported that FES-BM can better restore the motor function of the patient’s upper limbs than cyclic FES (Chan et al., 2009; Knutson et al., 2014, 2016) due to FES-BM combines FES and the bilateral symmetrical movement which has been proven to improve the patient’s motor function better than unilateral exercise training (Summers et al., 2004). This may be due to upper limbs are centrally linked as a coordinative structure unit (Bernstein, 1966) and mirror-symmetrical movements are the classic coordination modes in the human repertoire (Kelso, 1995).

Building an accurate mapping relationship from voluntary movement intention to FES can make the stimulated movement of patient’s affected limb more precise (Zhou et al., 2020) and improve the recovery (Schick et al., 2017). Kinematic information (Knutson et al., 2007; Ruiz-Olaya et al., 2019; Malesevic et al., 2021) has been used as the feedback for FES control in some studies. For example, Knutson et al. (2007) adjusted the amplitude of FES proportionally according to the bending angle of the finger. However, kinematic information cannot reflect the patient’s movement intention well when there are external forces such as grasping objects with different strengths. Thus, the control based on muscle force is considered superior to the control based on kinematic information.

Therefore, many efforts have been made to establish the relationship between surface electromyography (sEMG) signals and FES to generate the desired muscle force. Zhou et al. (2016) used the wrist torque during isometric contraction as an intermediate variable to the transformation from sEMG to the pulse width (PW) and frequency of FES for flexor carpi radialis (FCR) or extensor carpi ulnaris (ECU). They also used static force during grasp as an intermediate variable to determine the transfer relationship between sEMG features and FES parameters for flexor digitorum superficialis (FDS) (Zhou et al., 2020). In these studies, force generated during static isometric contraction was used as the intermediate variables due to that the sEMG–force relationship was easily affected by joint motion. However, the actual FES control was executed during dynamic tasks. The sEMG–FES parameter relationship obtained during static contractions was different from that in dynamic contractions. Therefore, to improve FES control for dynamic tasks, it is necessary to establish the sEMG–FES parameter relationship during dynamic tasks.

Joint torque is a dynamic information that can reflect motion intentions and forces (Huang et al., 2020). Joint torques calculated based on optical motion capture devices are considered to be the most accurate, but it is impossible to use optical capture devices in practical application. Therefore, some studies estimated joint torque through the musculoskeletal model (Hou et al., 2016; Chen et al., 2017). However, the musculoskeletal model contains many physiological parameters that are difficult to measure. Huang et al. (2020) estimated joint torque from sEMG and joint angle based on backpropagation neural networks and found that joint angles also played a role in the estimation process. It proved that joint angle, as an index to describe joint motion, can improve the accuracy of prediction of joint torque based on sEMG. Therefore, combining kinematic information and sEMG can better realize dynamic movement estimation. It may also be applicable to the estimation of FES parameters.

On the other hand, at present, most practical FES applications only stimulate one muscle (Xiao et al., 2018; Zhou et al., 2020), which is inconsistent with the neural strategies of human motor control, that humans recruit multiple muscles rather than a single muscle when performing movements. It has been largely suggested that our central nervous system (CNS) recruits a group of muscles in a fixed pattern to reduce the dimension of information processing, which is defined as muscle synergies (Bernstein, 1966; d’Avella et al., 2003; Ison and Artemiadis, 2014). Stimulating a group of muscles in this fixed pattern can mimic this physiological property. Additionally, biomimetic multi-channel FES systems have been proved to be beneficial for recovery (Ambrosini et al., 2011; Ferrante et al., 2016), and they could reduce muscle fatigue compared with single-channel FES (Happak et al., 1989; Wang et al., 2021). Moreover, it was found that the multi-channel FES could improve muscle coordination for patients (Ferrante et al., 2016; Niu et al., 2019).

The aim of this study was to develop a dynamic multi-channel mapping strategy from voluntary sEMG and joint motion to FES parameters. This strategy was used to achieve high symmetrical movements of bilateral hands. Compared with the previous studies, this strategy achieved a dynamic prediction during movement instead of during static contractions. To dynamically reproduce the target joint torque, the joint angle and mean absolute value (MAV) of sEMG were used to modulate the PW of FES through a proposed direct transfer function (DTF). This strategy was also applied to a real-time FES system to verify its online accuracy. We hypothesized that this strategy would improve the accuracy of FES-induced movement.

## Materials and Methods

### Subjects

A total of six healthy right-handed subjects (4 men and 2 women; aged: 21–25; referenced as S1–S6) were selected and invited to participate in the experiment. Any participant with a history of wrist injuries and wrist extension elicited by FES with pain was excluded from the study. All the subjects gave their written informed consent before the experiment. The study was approved by the Ethics Committee of Tianjin University.

### Experiment

#### Preliminary Experiment

Before the formal experiment, there were two preliminary experiments for each subject. The first one was performed to calculate the muscle synergy of each subject. The subjects were asked to sat naturally in the chair with their upper arms putting on the table in front of them. Then, two wireless sEMG sensors (Trigno™ Avanti Platform, DELSYS, United States), sampled at 2000 Hz, were placed in the direction of the muscle fibers of ECU and extensor carpi radialis (ECR) of right hand. Then, they were asked to keep wrist extension for 20 s and the sEMG data were recorded. The muscle synergy matrix W was calculated to set the ratio of each FES channel for each subject, which was described in section “Surface Electromyography Processing.”

The second preliminary experiment was performed to measure the threshold of the FES PW for each subject. The FES device was the RehaStim2 (HASOMED GmbH, Magdeburg, Germany) and the pulse can be controlled through the ScienceMode2 communication protocol (Kuberski, 2012). The value of PW of multi-channel FES was set based on muscle synergy. Then, two pairs of stimulating electrodes (3 cm × 3 cm) were placed on the muscle belly of ECU and ECR of left arm. The proportion of PW of each channel was based on the proportion of muscle synergy matrix W for each subject. The stimulation pulse waveform used for FES was a biphasic square-wave waveform with the amplitude of 15 mA and the frequency of 40 Hz for 2 s. The lower threshold of the PW, *PW*_*min*_, was determined by increasing the PW from 10 μs (frequency = 40 Hz, amplitude = 15 mA) with 10-μs increments until the subject’s wrist moved. The upper threshold of the PW, *PW*_*max*_, was determined by increasing the PW from 10 μs (frequency = 40 Hz, amplitude = 15 mA) with 10-μs increments until the subject had pain. The thresholds of PW of ECR and the ratio between PW of ECR and ECU for each subject are shown in Table 1.

**TABLE 1:** The thresholds of PW and the ratio between PW of ECR and ECU for subjects.

Subject	Frequency = 40 Hz, amplitude = 15 mA		Ratio
	PW_*min*_ (μs)	PW_*max*_ (μs)	
S1	120	290	0.698
S2	120	250	0.679
S3	170	270	0.815
S4	120	260	0.918
S5	150	300	0.779
S6	160	300	0.642

#### Formal Experiment

**FIGURE 1:**
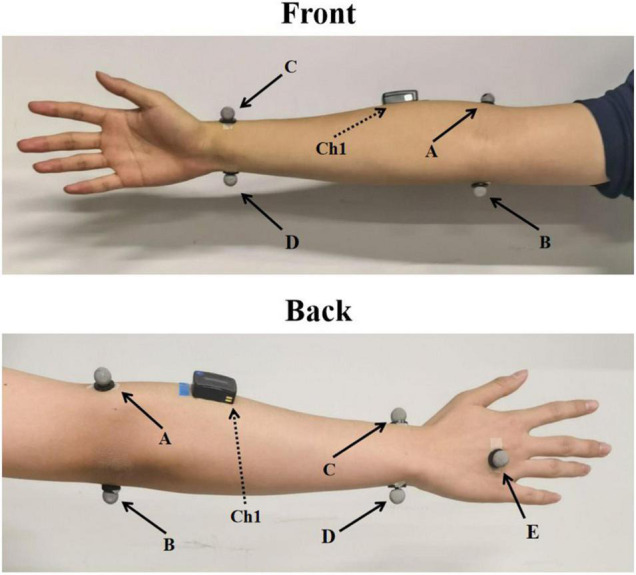
The placement of markers and sEMG sensor. A–E indicates the corresponding five markers and Ch1 indicates sEMG sensor placed at ECR.

In the formal experiment, there were three parts for each subject. A total of five reflective spherical markers were attached on bilateral hands and arms of each participant. The position of markers is shown in Figure 1. Kinematic data of the wrist movements were obtained simultaneously with EMG acquisition system from Vicon optical motion capture system (VICON VERO, OML (Oxford Metrics Limited), United Kingdom), sampled at 100 Hz. The marker data were filtered using a fourth-order Butterworth low-pass filter with the cutoff frequency of 10 Hz. Subjects sat comfortably in the chair and put both arms on the table in front of them. The first one was performed to build the transfer functions to map sEMG and joint torque. In this part, only one sEMG sensor was placed in the direction of the muscle fibers of ECR of right hand. The sEMG from ECR was widely used to estimate the motion intention of wrist (Khokhar et al., 2010; Song et al., 2013). They were asked to perform seven sessions of wrist extension using their right hand at random speed (the wrist extension time was fixed to 2 s) with sEMG from ECR and kinematic data were recorded simultaneously. Each session included five trails of wrist extension, and there was 1-min rest between each session. Each subject performed 35 trails of wrist extension movements in total. The first six sessions of data were used to establish the transfer function mapping the joint angle and MAV to the joint torque. The inputs of the transfer function were the MAV and the joint angle and the output was the joint torque, which was described in section “Polynomial Fitting.” The last session of data was used to verify the accuracy of the transfer function.

The second part was performed to build the transfer functions to map FES and joint torque. In this part, two pairs of stimulating electrodes (3 cm × 3 cm) were placed on the muscle belly of ECU and ECR of left arm. The FES PW of ECR increased from *PW*_*min*_ (frequency = 40 Hz, amplitude = 15 mA) with 10-μs increments was used to stimulate the subjects to perform wrist extension until PW was equaled to *PW*_*max*_. During the stimulation, the subjects did not perform voluntary contractions. The kinematic data were recorded simultaneously, and there was a 1-min rest between stimulations to avoid the effects of muscle fatigue. The kinematic data and the value of PW were used to establish the transfer function mapping the joint angle and joint torque to PW. The inputs of the transfer function were the joint torque and angle and the output was the PW, which was described in section “Polynomial Fitting.”

The third part was performed to verify the accuracy of the DTF. Based on the data of the above two parts, the DTF of the subjects can be established. The last session of sEMG and kinematic data from the voluntary movement was inputted into the DTF, and the estimated PW was the output. The estimated PW was used to stimulate the left arms of subjects with kinematic data recorded simultaneously. The FES-induced joint torque was compared with the voluntary joint torque to analyze the accuracy of the FES-induced joint torque of the transfer functions. The joint torque was calculated from the kinematic data based on the inverse dynamics, and the method was described in section “Joint Angle.” It should be noted that only the wrist extension movement phase was intercepted for training and analysis, because only extensor muscles were recorded and stimulated. The wrist extension movement phase was intercepted between the start of movement and 110° wrist angle. The start of movement was the crossing time when the angular velocity of the wrist joint crossed the threshold *mean*(ω_*rest*_) + 3*sd(ω_*rest*_) from bottom to top and maintained over the threshold for more than 30 ms, where ω_*rest*_ indicated the wrist angular velocity at rest, and *mean*(ω_*rest*_) and *sd*(ω_*rest*_) indicated the mean and standard deviation of ω_*rest*_.

### Surface Electromyography Processing

The sEMG signal data were filtered using a fourth-order Butterworth band pass filter between 20 and 300 Hz and a notch filter with 50 Hz and its frequency multiplication. Then, the feature MAV was calculated from sEMG the signal by Equation 1.


(1) $$M\,A\,V_{i}=\frac{1}{N}\,\sum_{k=0}^{N-1}\left|E_{i-k}\right|$$

where *MAV*_*i*_ is the MAV at the ith point of the sEMG data, *E*_*i–k*_ is the sEMG data after preprocessing at the i − kth point, and N is the length of the window. In this paper, the length of the window was 200 samples (100 ms) and the MAV was calculated with a sliding window of 20 samples (10 ms) to make sure that the MAV and the kinematic data can correspond one-to-one according to time.

The non-negative matrix factorization (NMF) algorithm was used to extract the features of muscle synergy. Muscle activation pattern can be calculated as follows:


(2) $$\begin{array}{c} E=W_{1}\cdot H_{1}+W_{2}\cdot H_{2}+\cdots +W_{k}\cdot H_{k}+e\,r\,r\,o \end{array}$$

where *E* is the m × n sEMG data set matrix (the number channels of sEMG signals, n is the signal length), *W*_*k*_ is the kth m × 1 matrix of synergy, *H*_*k*_ is the kth 1 × n matrix of time-varying synergy activation coefficient, and k is the number of synergies. W represents the activation degree of each muscle. In this paper, the number of synergies was 1 and W is normalized by dividing by the maximum value of element in W. Due to the number of muscles involved in wrist extension is limited and the difficulty to determine the FES PW when the number of muscle synergies increased, only two sEMG channels and one muscle synergy were used in this study.

### Kinematic and Kinetic Data Processing

#### Joint Angle

**FIGURE 2:**
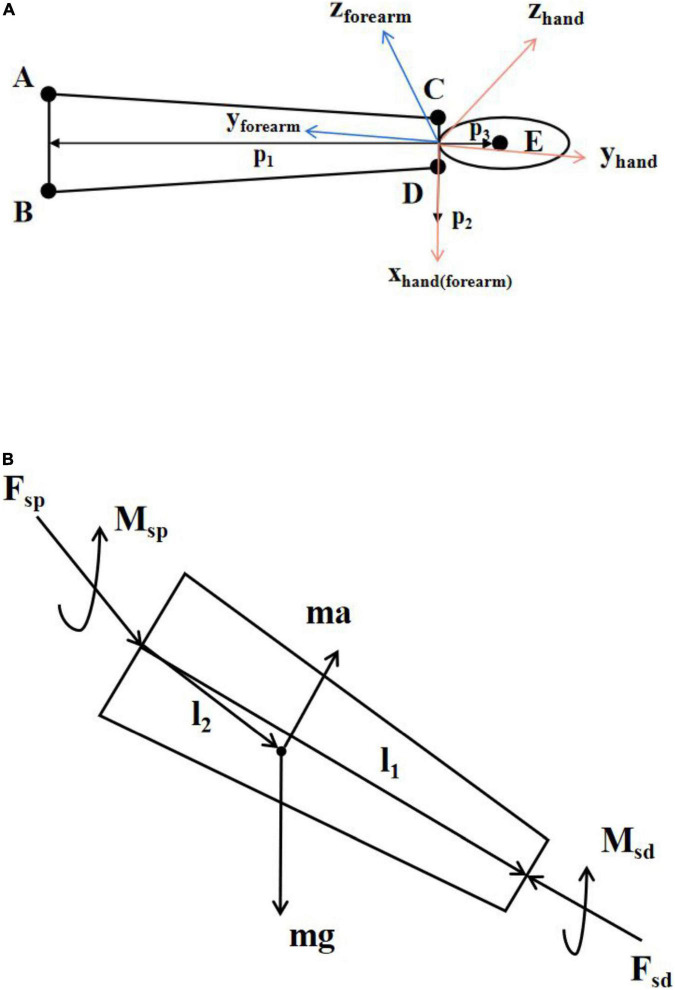
**(A)** Simplified model of hand and forearm. A–E indicates the corresponding five markers. The orange coordinate system is the hand coordinate system and the blue coordinate system is the forearm coordinate system. The *x*-axis of the hand coordinate system and the forearm coordinate system are the same. **(B)** Schematic diagram of inverse kinetics.

We built a simplified model of the wrist joint. In the Vicon coordinate system, we defined the direction of the vector *p*_1_ along the line connecting the midpoint of point A and point B and the midpoint of point C and point D, the direction of the vector *p*_2_ along the line connecting point C and point D and the direction of the vector *p*_3_ along the line connecting point E and the midpoint of point C and point D as shown in Figure 2A. The vector *p*_1_ and the vector *p*_2_ determined the plane of the forearm and the vector *p*_2_ and the vector *p*_3_ determined the plane of the hand.

Taking the vector p_2_ as the *x*-axis, the local coordinate system of the forearm (R_*f*_ = [*e*_*fx*_, *e*_*fy*_, *e*_*fz*_]) and hand (R_*h*_ = [*e*_*hx*_, *e*_*hy*_, *e*_*hz*_]) can be established as follows:


(3) $$\{\begin{array}{c} \begin{array}{l} e_{fx}=e_{hx}=\frac{p_{2}}{\left|p_{2}\right|} \end{array}\\ \begin{array}{l} e_{fz}=\frac{p_{1}\times p_{2}}{\left|p_{1}\times p_{2}\right|}\; \end{array}\\ \begin{array}{l} e_{fy}=e_{fx}\times e_{fy}\; \end{array}\\ \begin{array}{l} e_{hz}=\frac{p_{2}\times p_{3}}{\left|p_{2}\times p_{3}\right|}\; \end{array}\\ e_{hy}=e_{hx}\times e_{hz} \end{array}$$

where × is the vector product.

The rotation matrix *R*_*hf*_ between the forearm’s local coordinate system and the hand’s coordinate system can be calculated as follows:


(4) $$\begin{array}{c} R_{h\,f}={\left(R_{h}\right)}^{-1}\,R_{f} \end{array}$$

The joint angle θ_*w*_, the joint angular velocity ω_*w*_, and the joint angular acceleration α_*w*_ between the hand and the forearm in the direction of wrist flexion and extension can be calculated as follows:


(5) $$\begin{array}{c} \left\{ \;\begin{array}{c} \theta_{w}=t\,a\,n\,h^{-1}\,\left(-\frac{R_{h\,f}\,\left(2,3\right)}{R_{h\,f}\,\left(3,3\right)}\right)\\ \omega_{w}=\frac{d\,\theta_{w}}{d\,t}\\ \alpha_{w}=\frac{d\,\omega_{w}}{d\,t} \end{array}\right. \end{array}$$

#### Joint Torque

Inverse dynamic is a common method for kinetic analysis of human motion (Hatze, 2002; Riemer et al., 2008; Arteaga et al., 2020). It calculates the resultant forces and torques at both ends of the body segment from the inertia and kinematic information. The forces and motion parameters on the body segment are shown in Figure 2B, where m is the mass of the body segment, a is the acceleration of the body segment, g is the acceleration of gravity, *F*_*sd*_ and *M*_*sd*_ are the force and torque at the distal end of the body segment, *F*_*sp*_ and *M*_*sp*_ are the force and torque at the proximal end of the body segment, and *l*_1_ and *l*_2_ are the distance vector from the rotation center of the proximal joint of the body segment to the rotation center of the distal joint of the body segment and the distance vector from the center of rotation of the proximal joint of the body segment to the center of mass.

According to Newton–Euler equations, the dynamic equation of the body segment can be established as follows:


(6) $$\{\begin{array}{l} \begin{array}{l} F_{sp}+F_{sd}+mg=ma \end{array}\\ M_{sp}+M_{sd}-l_{2}\times F_{sp}+(l_{1}-l_{2})\times F_{sd}=I\text{α}+\text{ω}\times I\text{ω} \end{array}$$

where α is the angular acceleration of body segment, ω is the angular velocity of body segment, *I* is the moment of inertia of the body segment, and × is the vector product. The force on the distal end of the hand is zero during wrist extension. The mass, center of mass, and moment of inertia of the hand can be estimated from the human body parameters (De Leva, 1996; Dumas et al., 2004). Table 2 lists the subjects’ physiological data. Therefore, the joint torque M_*w*_ of the wrist during wrist extension can be calculated as follows:


(7) $$\begin{array}{c} M_{w}=l_{2}\times \left(m_{h}\,a_{h}-m_{h}\,g\right)+I_{h}\,\alpha_{w}+\omega_{w}\times I_{h}\,\omega_{w} \end{array}$$

**TABLE 2:** Physiological data of subjects.

Subject	S1	S2	S3	S4	S5	S6

Gender	m	m	m	f	f	m
Mass of body/kg	77.6	70.2	73.0	53.5	51.8	77.8
Length of hand/m	0.174	0.172	0.165	0.152	0.156	0.179
I (1,1) of hand/kg × m^2^	0.0023	0.0020	0.0019	0.0014	0.0012	0.0024
I (2,2) of hand/kg × m^2^	0.0038	0.0033	0.0032	0.0023	0.0020	0.0040
I (3,3) of hand/kg × m^2^	0.0057	0.0050	0.0048	0.0035	0.0030	0.0060
Center of mass of hand/m (distance from wrist)	0.0637	0.0630	0.0604	0.0576	0.0544	0.0655

*I (1,1), I (2,2), and I (3,3) are the values of the elements on the diagonal line of the moment of inertia of the body segment (I).*

where *m*_*h*_ is the mass of the hand, *a*_*h*_ is the acceleration at the center of mass of the hand, *I*_*h*_ is the moment of inertia of the hand, and × is the vector product.

### Polynomial Fitting

**FIGURE 3:**
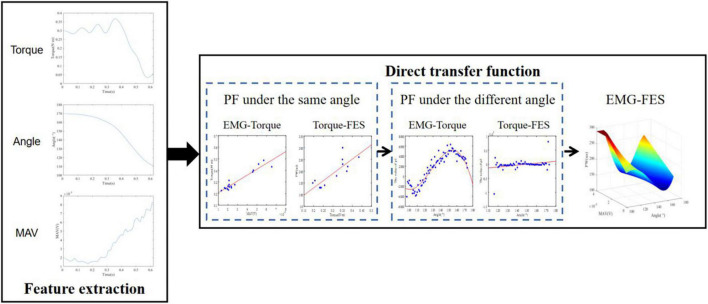
The FES estimation procedure.

We built a DTF of EMG, joint angle, and FES through polynomial fitting (PF) as shown in Figure 3. The transfer function is subject-specific due to the factors such as resistance between skin and electrode, electrode location, and fat thickness.

**FIGURE 4:**
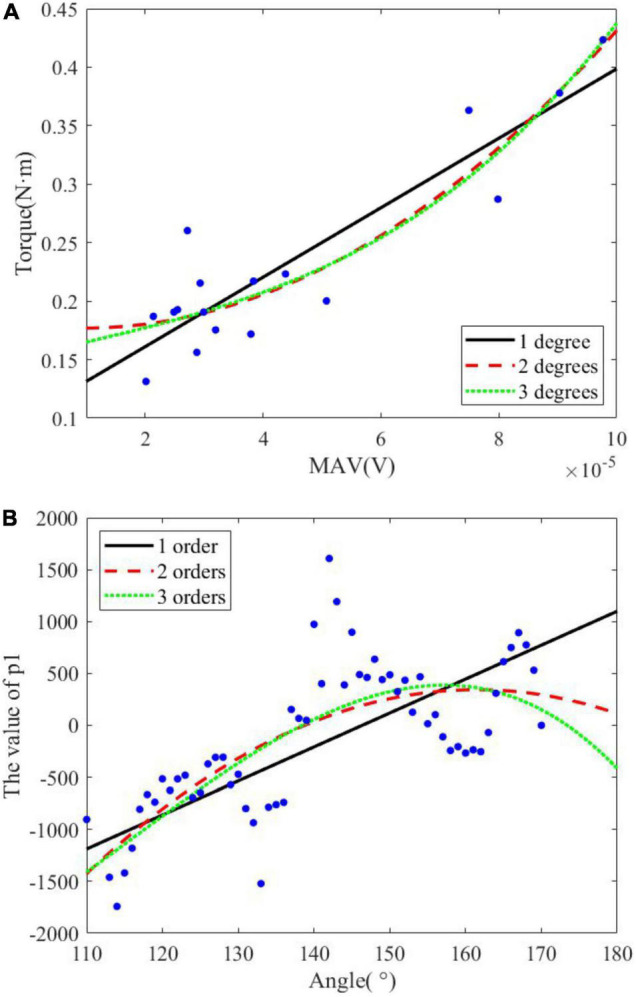
Polynomial fitting at different degrees. **(A)** Example of the fitting of MAV and joint torque. **(B)** Example of the fitting of *p*_1,θ_ and joint angle.

It has been proved that the wrist position affects sEMG signal (Roman-Liu and Bartuzi, 2013; Jochumsen et al., 2018) and FES may also be affected. A polynomial transfer function was established to map MAV, joint torque, and PW at each integer angle firstly, which could be considered to describe the relationship at isometric contraction. The joint angle was approximated to an integer to ensure that there were enough samples for each angle to fit. Dozens of polynomial transfer functions for wrist extension were obtained. Figure 4A shows an example of the fitting of MAV and joint torque at different fitting orders at 135°. The polynomial transfer function mapping MAV to the estimated joint torque and that mapping the actual joint torque at each integral angle were listed as Equation 8.


(8) $$\{\;\begin{array}{l} \begin{array}{l} T_{i}=p_{1,i}MAV_{i}{}^{n}+p_{2,i}MAV_{i}{}^{n-1}+\cdots +p_{n,i}MAV_{i}+p_{n+1,i}\; \end{array}\\ PW_{i}=q_{1,i}T_{i}{}^{n}+q_{2,i}T_{i}{}^{n-1}+\cdots +q_{n,i}T_{i}+q_{n+1,i} \end{array}$$

where *MAV*_*i*_, *PW*_*i*_, and *T*_*i*_ are the estimated joint torque, the value of MAV, the PW of FES, and the actual joint torque at i° and n is the fitting orders of *MAV*_*i*_ and *T*_*i*_. *p*_*n,i*_ and *q*_*n,i*_ are the coefficients of the two polynomial functions at i°, respectively. At different wrist positions, polynomial functions had different coefficients. Therefore, joint angles and coefficients at different angles were fitted. To ensure enough data for fitting, we consider all the angles × around the integer angle A (A − 0.5 ≤ × < A + 0.5) to be the same isometric contraction state, which can be used to establish the fitting function at A. The range of angles to establish the polynomial transfer functions depended on the angular range of wrist extension for each subject. Figure 4B shows an example of the fitting of *p*_*1,θ*_ and joint angle at one order of *T*_*i*_. The coefficients at different angles can be calculated by Equations 9, 10.


(9) $$\begin{array}{c} \left\{ \begin{array}{c} p_{1,\theta }=f_{1}\,\left(\theta \right)=a_{1,1}\,\theta^{m}+a_{2,1}\,\theta^{m-1}+\cdots \\ +a_{m,1}\,\theta +a_{m+1,1}\\ \vdots \\ p_{n+1,\theta }=f_{n+1}\,\left(\theta \right)=a_{1,n+1}\,\theta^{m}+a_{2,n+1}\,\theta^{m-1}+\cdots \\ +a_{m,n+1}\,\theta +a_{m+1,n+1} \end{array}\right. \end{array}$$

(10) $$\begin{array}{c} \left\{ \begin{array}{c} q_{1,\theta }=g_{1}\,\left(\theta \right)=b_{1,1}\,\theta^{m}+b_{2,1}\,\theta^{m-1}+\cdots +b_{m,1}\,\theta \\ +b_{m+1,1}\\ \vdots \\ q_{n+1,\theta }=g_{n+1}\,\left(\theta \right)=b_{1,n+1}\,\theta^{m}+b_{2,n+1}\,\theta^{m-1}+\cdots \\ +b_{m,n+1}\,\theta +b_{m+1,n+1} \end{array}\right. \end{array}$$

where θ is the joint angle and *m* is the fitting orders of θ. *a*_*m,n*_ and *b*_*m,n*_ are the coefficients of the two polynomial functions, respectively.

The DTF of PW, MAV, and joint angle can be obtained by combining the polynomials obtained by fitting as follows:


(11) $$\begin{array}{c} \{\;\begin{array}{l} PW_{\text{θ}}=g_{1}\left(\text{θ}\right){\left(T\left(MAV_{\text{θ}}\right)\right)}^{n}+g_{2}\left(\text{θ}\right){\left(T\left(MAV_{\text{θ}}\right)\right)}^{n-1}+\cdots \\ \begin{array}{l} \;+g_{n}\left(\text{θ}\right)T(MAV_{\text{θ}})+g_{n+1}\left(\text{θ}\right)\; \end{array}\\ T\left(MAV_{\text{θ}}\right)=f_{1}\left(\text{θ}\right)MAV_{\text{θ}}{}^{n}+f_{2}\left(\text{θ}\right)MAV_{\text{θ}}{}^{n-1}+\cdots \\ \;+f_{n}\left(\text{θ}\right)MAV_{\text{θ}}+f_{n+1}\left(\text{θ}\right) \end{array} \end{array}$$

### Experiment of Real-Time Functional Electrical Stimulation

To understand how this strategy performed in a real-time FES system, an online experiment of FES combined with the DTF was designed. S1 and S4 took part in the experiment. The real-time FES-BM system in the study of Zhao et al. (2021) was used in this study. The wrist joint angle was calculated by two inertial measurement units (IMUs) (Trigno™ Avanti Platform, DELSYS, United States), which were placed at the right hand and forearm, respectively. Each subject was asked to put both their arms on the table in front of them with the same initial wrist angle. They were asked to perform five wrist extensions in one session, and there were three sessions in total. During the extension, FES parameters were generated according to the collected EMG and kinematic data of the right hand and used to stimulate the left hand to induce movement. The real-time status was shown and recorded by a GUI designed by LabVIEW (National Instruments, Inc.). The kinematic data were recorded by Vicon to verify the accuracy in real time.

### Evaluation

The orders of MAV or T and the orders of θ for PF were investigated to get the optimal fitting. They were developed from one to three orders. Therefore, there were nine combinations for transfer functions. The coefficient of determination (*R*^2^) of estimated joint torque and true joint torque was used as an indicator for comparison of the transfer functions fitting MAV and joint angle to joint torque of each combination. The *R*^2^ of estimated PW and true PW was used as an indicator for comparison of the transfer functions fitting joint torque and joint angle to PW of each combination. A one-way repeated measures analysis of variance (ANOVA) was applied to investigate the effect of the degrees of MAV/T and the degrees of θ for PF.

The accuracy of joint torque estimation affected the accuracy of PW estimation and the accuracy between the FES-included joint torque and the voluntary joint torque reflected the effectiveness of the strategy. Therefore, *R*^2^ and the normalized root mean square error (NRMSE) of joint torque and estimated joint torque were used as an indicator to verify the accuracy of estimation. Moreover, *R*^2^ and NRMSE of FES-included joint torque/angle and the voluntary joint torque/angle were used as an indicator to verify the accuracy of DTF.

## Results

### Muscle Synergy

**FIGURE 5:**
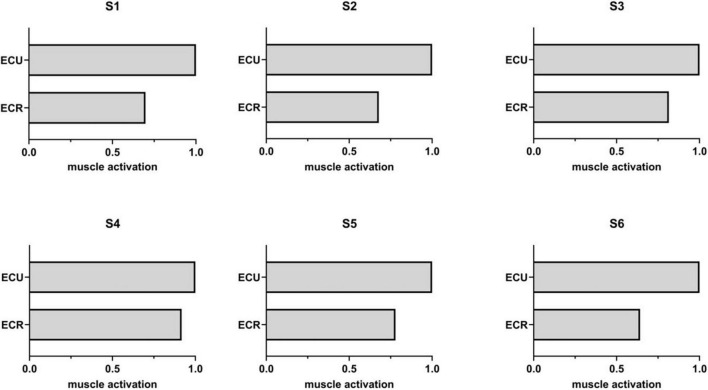
The synergy matrix for all subjects during wrist extension.

Figure 5 shows the synergy matrix of all subjects during wrist extension. It can be seen that the synergy matrix was similar for all subjects, which revealed the reliability of synergy theory. When the number of synergies was 1, *R*^2^ = 0.773 ± 0.068 and variance accounted for (VAF) (Israely et al., 2018) was 0.792 ± 0.093. It showed that the factorization was accurate, although the number of synergies was 1.

### Fitting Order Selection for Polynomial Fitting

**FIGURE 6:**
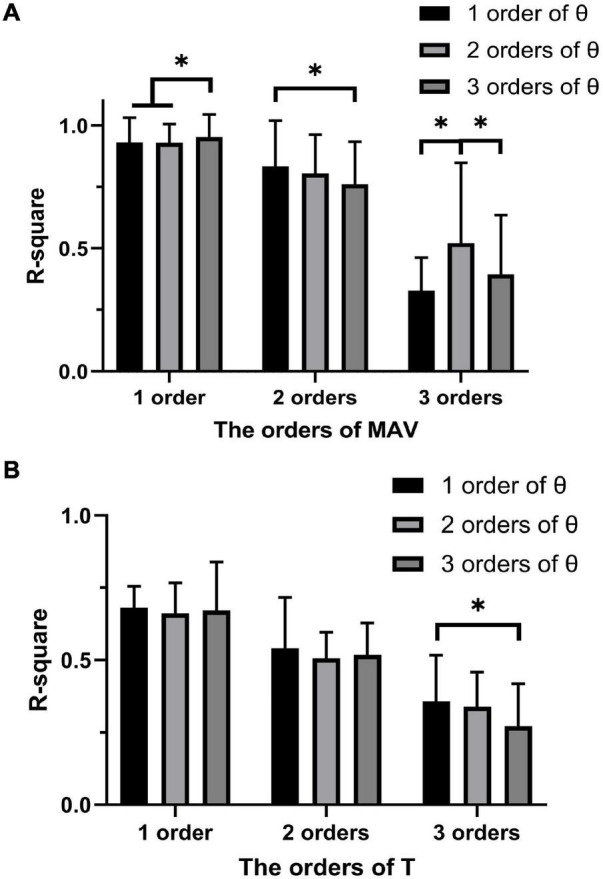
**(A)** The average accuracy for PF of MAV and joint angle at different degrees. **(B)** The average accuracy for PF of joint torque and joint angle at different degrees. The symbol “*” indicates a significant difference (*p* < 0.05).

Figure 6A shows the *R*^2^ for PF of all subjects between the joint toque, MAV and joint angle. *R*^2^ of one-order PF for MAV was significantly higher than that of two-order PF for MAV (*p* < 0.001) and three-order PF for MAV (*p* < 0.001). For the one-order PF for MAV, *R*^2^ of three-order PF for θ was significantly higher than that of one-order (*p* = 0.004) and two-order (*p* < 0.001) PF for θ. Therefore, the combination of one-order PF for MAV and three-order PF for θ were selected as the model of PF to build the relationship between the joint toque, MAV, and joint angle. The combination of one-order PF for MAV and three-order PF for θ had the best accuracy (*R*^2^ = 0.952 ± 0.092).

Similarly, Figure 6B shows the *R*^2^ for PF of all subjects between the PW, joint torque, and joint angle. The ANOVA analysis revealed that no significant difference appeared between the one-order, the two-order, and the three-order PF for T, but the combination of one-order PF for joint torque and one-order PF for joint angle has less variability and a higher *R*^2^ (*R*^2^ = 0.681 ± 0.075). Therefore, the combination of one-order PF for joint torque and one-order PF for joint angle was selected as the model of PF to build the relationship between the PW, joint torque, and joint angle.

### The Accuracy of Joint Torque Estimation

**FIGURE 7:**
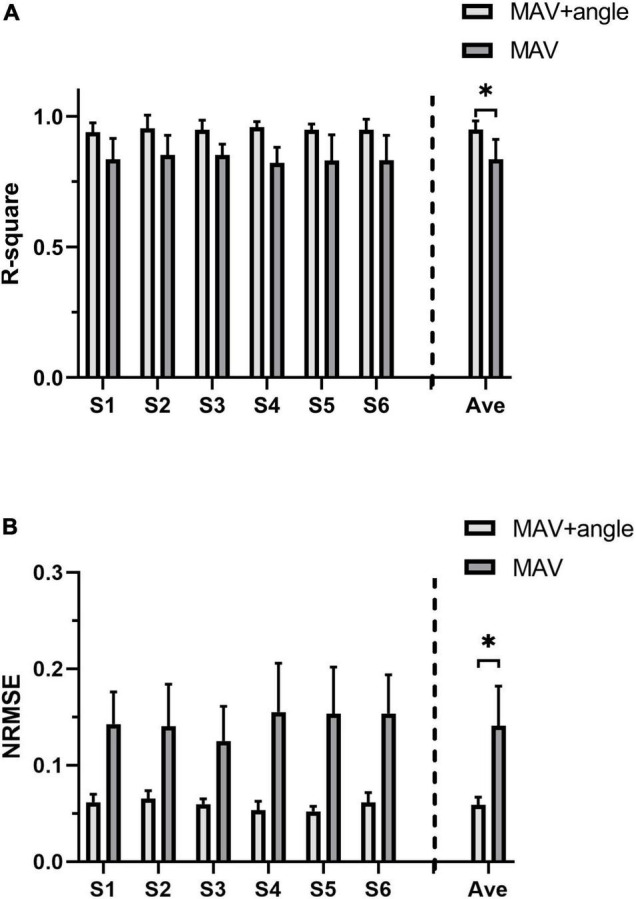
The joint torque estimation accuracy of all subjects. **(A)** *R*^2^ of joint torque estimation based on MAV and joint angle and joint torque estimation based on MAV only. **(B)** NRMSE of joint torque estimation based on MAV and joint angle and joint torque estimation based on MAV only. The symbol “*” indicates a significant difference (*p* < 0.05).

**FIGURE 8:**
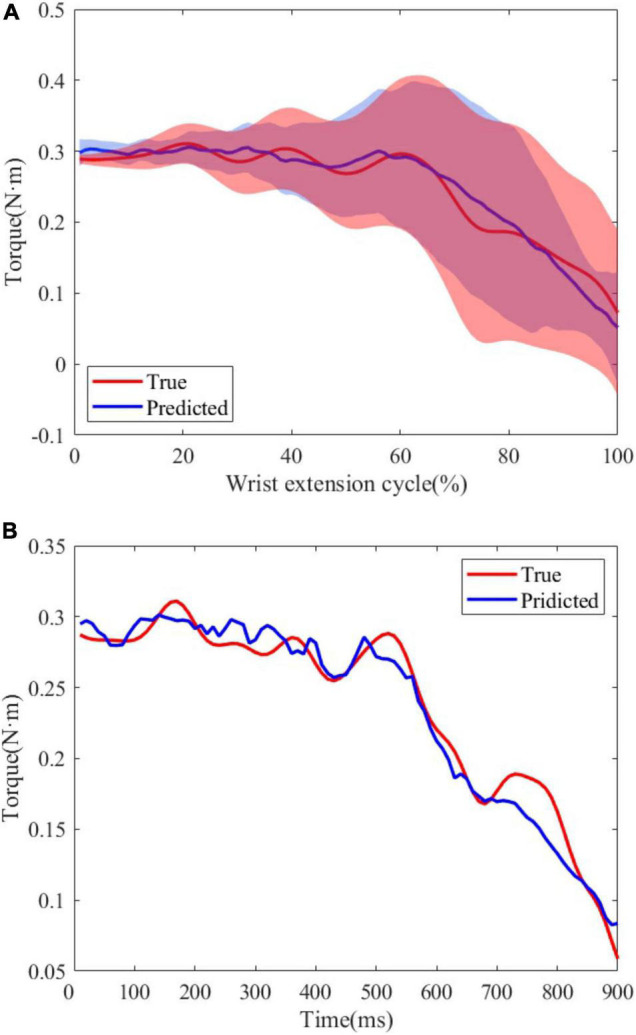
**(A)** True and predicted joint torque based on MAV and joint torque for all subjects. The shadow regions represent the standard deviations and the lines represent the average joint torque for all subjects. **(B)** A representative trial of predicted joint torque of S1.

*R*^2^ and NRMSE of joint torque estimation of all subjects are shown in Figure 7. The coefficient of determination was 0.949 ± 0.033 (max: S4: *R*^2^ = 0.954 ± 0.050, min: S6: *R*^2^ = 0.939 ± 0.036) and the NRMSE was 0.059 ± 0.008 (max: S2: NRMSE = 0.065 ± 0.008, min: S5: NRMSE = 0.052 ± 0.005). Figure 8A shows true and predicted joint torque based on MAV and joint torque for all subjects and Figure 8B shows a representative trial of predicted joint torque of S1, where the time of each wrist extension was normalized. We also built a three-order polynomial transfer function mapping MAV to joint torque to demonstrate the importance of joint angle. *R*^2^ and NRMSE of joint torque estimation based on MAV only were 0.836 ± 0.106 and 0.141 ± 0.041, respectively, as shown in Figure 7, which was significantly lower (*p* < 0.001) than the function combined with MAV and joint angle.

### The Accuracy of Functional Electrical Stimulation-Included Joint Torque/Angle

The last session of sEMG and kinematic data was used to verify the accuracy of the transfer function for each subject. Due to the presence of the PW threshold for each subject, the estimated value beyond the threshold was set to the lower or upper threshold. At the same time, the minimum interval of PW in Rehastim2 FES device was 10 μs and the frequency of FES was 40 Hz. Therefore, the PW was changed every 25 ms and the PW value used for stimulation was a 10-digit integer that rounded the mean of the estimated PW values 25 ms before.

**FIGURE 9:**
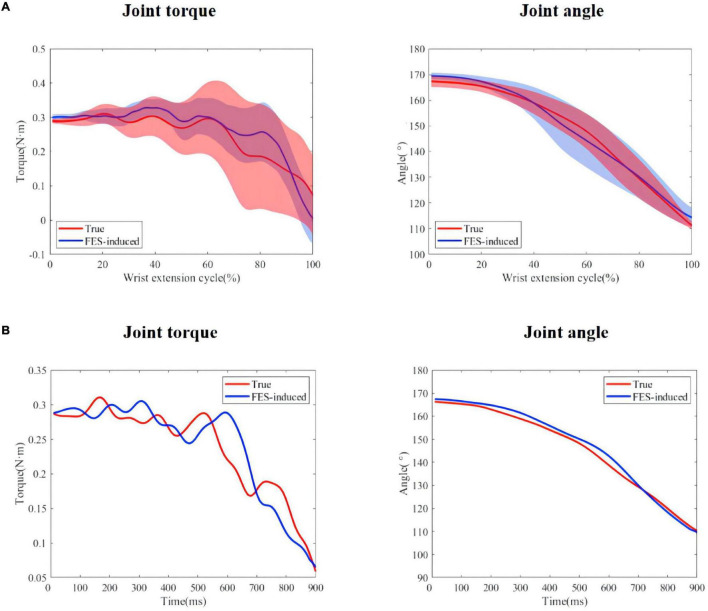
**(A)** True and predicted FES-included movement of S1. The shadow regions represent the standard deviations and the lines represent the average joint torque/angle during the five stimulation trials. **(B)** A representative trial of FES-included movement of S1.

The same length data was intercepted from the moment when the wrist extension started to compare the similarity between the FES-included movement and the voluntary movement. Table 3 shows the mean *R*^2^ and NRMSE of PF mapping the joint angle to the joint torque of each subject between the FES-induced and the voluntary wrist extension. The coefficient of determination and NRMSE for joint torque fitting were 0.701 ± 0.220 and 0.241 ± 0.080. The *R*^2^ and NRMSE of joint angle were 0.966 ± 0.042 and 0.047 ± 0.005. Figure 9A shows the true and FES-induced joint torque and joint angle of all subjects and Figure 9B shows a representative trial of FES-included movement of S1, where the time of each wrist extension was normalized.

**TABLE 3:** The coefficient of determination and NRMSE of the FES-induced and voluntary wrist extension.

Subject	Joint torque		Joint angle	
	*R* ^2^	NRMSE	*R* ^2^	NRMSE
S1	0.72088 ± 0.21260	0.22045 ± 0.06755	0.97606 ± 0.01851	0.04351 ± 0.00413
S2	0.81741 ± 0.07079	0.17548 ± 0.06259	0.99041 ± 0.00581	0.01220 ± 0.00133
S3	0.58755 ± 0.31304	0.30479 ± 0.10263	0.95180 ± 0.03253	0.05492 ± 0.00529
S4	0.74325 ± 0.10946	0.21252 ± 0.05987	0.97031 ± 0.04859	0.05027 ± 0.00547
S5	0.66418 ± 0.35803	0.27560 ± 0.09624	0.95793 ± 0.03974	0.06017 ± 0.00441
S6	0.67327 ± 0.15849	0.25647 ± 0.07432	0.94823 ± 0.07493	0.06373 ± 0.00692
Mean	0.70109 ± 0.22031	0.24089 ± 0.07963	0.96579 ± 0.04171	0.04747 ± 0.00473

### The Effect of Speed on Accuracy

**FIGURE 10:**
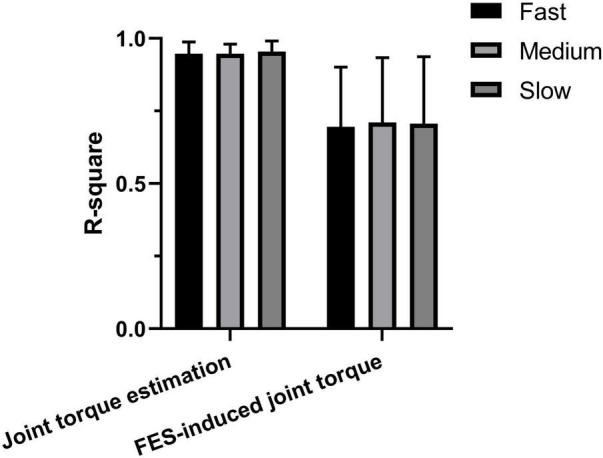
The accuracy of joint torque estimation with sEMG and joint torque production with FES at different speeds.

As the extension speed may affect the estimation accuracy, according to the t_110_ which represented the time from the start to wrist extension to joint angle of 110°, we divided the trials into three groups: fast (t_110_ < 400 ms), medium (400 ms < t_110_ < 800 ms), and slow (t_110_ > 800 ms). Figure 10 shows the *R*^2^ of joint torque and FES-induced joint torque of three groups. The *R*^2^ of joint torque estimation of fast, medium, and slow groups was 0.695 ± 0.206, 0.711 ± 0.224, and 0.707 ± 0.231, respectively. The *R*^2^ of FES-induced joint torque of fast, medium, and slow groups was 0.947 ± 0.041, 0.947 ± 0.033, and 0.954 ± 0.037, respectively. However, there was no significant difference between the *R*^2^ of joint torque estimation and FES-induced joint torque of three groups.

### The Accuracy of Functional Electrical Stimulation-Included Joint Torque/Angle in Real Time

**FIGURE 11:**
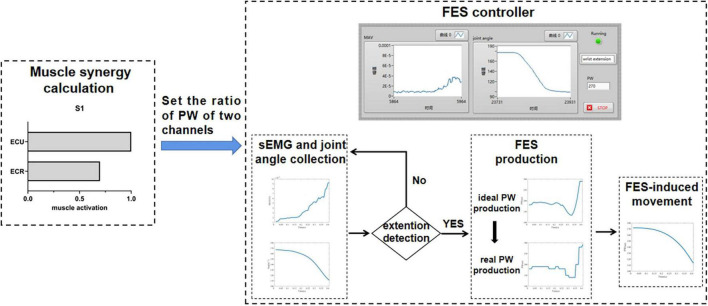
The design of the real-time FES system combined with the DTF.

**FIGURE 12:**
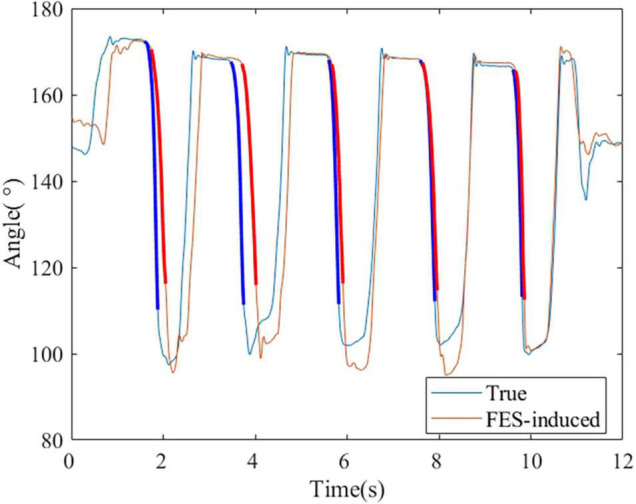
A representative session of S4. The bold lines indicate the wrist extension and FES-induced parts that were used to calculate the accuracy.

Figure 11 shows the process of the real-time FES system combined with the DTF. The ratio of PW of two FES channels was set based on the ratio obtained from muscle synergy, and the FES was triggered when a wrist extension was recognized. Figure 12 shows a representative session of S4. The latency of this system was between 30 and 200 ms. The *R*^2^ and NRMSE of FES-induced joint angle and joint torque were *R*^2^ = 0.940 ± 0.038 and NRMSE = 0.071 ± 0.014 and *R*^2^ = 0.607 ± 0.294 and NRMSE = 0.303 ± 0.119, respectively.

## Discussion

This study developed a dynamic multi-channel mapping strategy to improve the accuracy of desired force and the symmetry of bilateral movements during FES-BM, which has been proved to improve the rehabilitation effect of FES. The main contribution was that this study succeeded in estimating FES parameters based on joint angle and sEMG during dynamic tasks. Real-time estimation of FES parameters was realized with joint torque as the intermediate variable. This strategy not only reduced the influence of the joint angle on the EMG signal, but also avoided the influence of the movements. First, the contractions around each integer joint angle were regarded as the same isometric state, and the corresponding PF transfer function was calculated. Then, the PF transfer function mapping joint angle to each polynomial coefficient was established. The DTF realized the transformation from the MAV and joint angle to PW directly. Estimation of FES parameters based on transfer functions can reduce the computational complexity of the system and thus reduce the delay.

Muscle synergy has been used to control multi-channel FES in many studies. Previous studies show the similarities in muscle synergy between individuals (Overduin et al., 2008). Therefore, it is also feasible for FES control based on other people’s muscle synergy patterns (Niu et al., 2019). To improve the accuracy of FES-induced movement, Zhuang et al. (2015) applied muscle synergies to the virtual arm model to test a synergy based NMES strategy, and the results showed close resemblance to the original elbow trajectory of normal movements. Razavian et al. (2018) designed a muscle synergy-based FES controller which employed direct relations between the muscle synergies and the produced hand forces and achieved a final hand position error of 2 cm for a 2-D point-to-point reaching task. The previous studies usually used the muscle synergies W, which reflect the muscle pattern, as the FES pattern and the activation coefficients H, which reflected the sEMG activation, as the FES activation. Therefore, we used the muscle synergy to determine the ratio of the PW of two stimulation channels. With the use of muscle synergy, we controlled multi-channel FES based on only one sEMG channel. Unlike previous studies, our study replaced the activation coefficients H by the DTF, which can better fit voluntary movement and FES-induced movement. However, it was difficult to establish the DTF when the number of muscle synergies was not one. The number of muscle synergy was fixed one in our study. Our study proved that multi-channel FES based on muscle synergy could be used to control FES-induced torque.

For the estimation of joint torque, it was found that the combination of 1° PF for MAV and 3° PF for θ had the best fit. The accuracy of joint torque estimation for the transfer function based on joint angle and MAV (*R*^2^ = 0.949 and NRMSE = 0.059) was significantly higher than the transfer function based on MAV only (*R*^2^ = 0.836 and NRMSE = 0.141), which further demonstrated the advantages of combining sEMG and joint angle. Compared to the previous studies (Kamavuako et al., 2013; Yu et al., 2021), this study achieved a higher accuracy of wrist joint torque estimation with less sEMG electrodes by the combination of sEMG and kinematic information. Gregory found that there was a relationship between FES-induced torque and total charge (Gregory et al., 2007), which was also demonstrated in this paper. But few studies established the transfer function between the joint torque and PW. Similar to establishing the transfer relationship between joint torque and MAV, this study used PF to build the model. The combination of 1° PF for joint torque and 1° PF for θ had the best fit. However, the accuracy of PW estimation for PF model was only 0.681. It can be seen that the non-linear degree of PW and joint torque was not easy to be fitted by PF.

In terms of joint angle reproduction, this study achieved a high accuracy (*R*^2^ = 0.965 and NRMSE = 0.047). Rossi et al. (2020) proposed an average threshold crossing (ATC) FES control strategy, and the correlation coefficient of joint angle during elbow flexion was 0.87 ± 0.07. Bi et al. (2020) designed a multiple-gesture FES system, and the correlation coefficient of joint angle during wrist extension was 0.89 ± 0.04. The accuracy of FES-induced joint angle for this study was much better than previous studies. However, it was more difficult to generate desired joint torque. The accuracy of FES-induced joint torque was *R*^2^ = 0.701 and NRMSE = 0.241, which was lower than the FES-induced static force at isometric contraction in previous studies (the correlation coefficient, *R* = 0.912 ± 0.055 and *R* = 0.91 ± 0.04) (Zhou et al., 2016, 2020). Using FES to generate desired joint torque during movement is difficult, due to the effect of speed and position.

Building an accurate mapping relationship from desired force to FES is significant, especially in dynamic movement. Razavian et al. (2018) proposed a feedback controller for FES to control hand movements in a 2-D task space based on force. To our knowledge, our study is the first to propose a strategy for dynamic prediction of FES based on joint torque. The combination of sEMG and kinematic information can effectively reduce the influence of wrist position on sEMG signals. Moreover, it seems that the speed will not affect the accuracy of joint torque estimation and FES-induced joint torque during wrist extension.

When the control strategy was realized with a real-time system, the accuracy of FES-induced joint angle and joint torque was *R*^2^ = 0.940 ± 0.038 and NRMSE = 0.071 ± 0.014 and *R*^2^ = 0.607 ± 0.294 and NRMSE = 0.303 ± 0.119. The reason why the accuracy was lower than that of the offline method may be that the delay of the FES control system led to the inconsistency of the both wrist angles. Therefore, it is necessary to reduce the latency of gesture recognition and system communication to improve the accuracy of the real-time FES system combined with the DTF.

In this study, an estimation model of PW was established based on wrist extension, which are common in the rehabilitation of patients with stroke. High accuracy of joint angle and torque reproduction makes sure that the patients with stroke can simulate the force pattern of healthy muscles by themselves (Chan et al., 2009; Knutson et al., 2016) or experienced rehabilitation physicians (Huang et al., 2014; Zhou et al., 2016). In practical application, joint angles can be acquired using IMUs, data gloves, and bend sensors, etc.

However, the main purpose of this study was to evaluate the feasibility of building a relationship between joint angle, sEMG, and FES based on joint torque, and there are some limitations. First, the muscle fatigue and the discomfort from continuous movement have not been studied. Zhou et al. (2016) proposed that real-time controlled FES will reduce muscle fatigue produced by FES and the increasing of PW produced less discomfort than the increasing of FES amplitude. Therefore, PW was used as the FES parameters adjusted in this study. The muscle fatigue might affect the accuracy of joint torque reproduction. We will study the changes in accuracy and discomfort of movement during a continuous use in the future. Second, only healthy subjects were studied in this study. Although this strategy was established from the actual FES-induced joint torque which may reduce the effect of muscle atrophy of patients with stroke, the performance of this strategy still needs to be verified.

## Conclusion

In previous studies, the relationship between sEMG signal and FES control parameters was established during static isometric contraction. However, it is not appropriate to use the static relationship for FES control during movement. This study proposed a dynamic mapping strategy based on sEMG and kinematic information *via* joint torque. The coefficient of determination and NRMSE for FES-induced joint angle fitting were 0.965 and 0.047 and those for FES-induced joint torque fitting were 0.701 and 0.241. The DTF also had a good performance in real-time FES system, with *R*^2^ = 0.940 and 0.607 and NRMSE = 0.071 and 0.303 for the joint angle and torque fitting, respectively. It was concluded that the transfer function established under dynamic tasks *via* joint torque could achieve a good movement output for FES-BM. This FES control strategy estimated PW directly with a small amount of calculation and a short delay. Future work is still needed to test this strategy in patients with stroke and study the influence of muscle fatigue.

## Data Availability Statement

The raw data supporting the conclusions of this article will be made available by the authors, without undue reservation.

## Ethics Statement

The studies involving human participants were reviewed and approved by the Ethics Committee of Tianjin University. The patients/participants provided their written informed consent to participate in this study. Written informed consent was obtained from the individual(s) for the publication of any potentially identifiable images or data included in this article.

## Author Contributions

RX, XZ, ZW, and LM contributed to conception and design of the study. RX and XZ organized the database. RX, XZ, and ZW performed the statistical analysis. XZ wrote the first draft of the manuscript. RX, ZW, LM, and DM wrote sections of the manuscript. All authors contributed to manuscript revision, read, and approved the submitted version.

## Conflict of Interest

The authors declare that the research was conducted in the absence of any commercial or financial relationships that could be construed as a potential conflict of interest.

## Publisher’s Note

All claims expressed in this article are solely those of the authors and do not necessarily represent those of their affiliated organizations, or those of the publisher, the editors and the reviewers. Any product that may be evaluated in this article, or claim that may be made by its manufacturer, is not guaranteed or endorsed by the publisher.
